# Comparison of Three Intervention Models for Promoting Circumcision among Migrant Workers in Western China to Reduce Local Sexual Transmission of HIV

**DOI:** 10.1371/journal.pone.0076107

**Published:** 2013-09-30

**Authors:** Chuanyi Ning, Junjun Jiang, Li Ye, Xiaobo Yang, Bo Wei, Wei Deng, Suosu Wei, Jiegang Huang, Bo Qin, Halmurat Upur, Chaohui Zhong, Qianqiu Wang, Qian Wang, Yuhua Ruan, Fumei Wei, Na Xu, Peiyan Xie, Jenny H. Hsi, Yiming Shao, Hao Liang

**Affiliations:** 1 School of Public Health, Guangxi Medical University, Nanning, Guangxi, China; 2 Guangxi Key Laboratory of AIDS Prevention and Treatment, Guangxi Medical University, Nanning, China; 3 The First Affiliated Hospital, Chongqing Medical University, Chongqing, China; 4 School of Public Health, Xinjiang Medical University, Urumchi, Xinjiang, China; 5 School of Public Health, Chongqing Medical University, Chongqing, China; 6 National Center for STD Control, Chinese Center for Disease Control and Prevention, Nanjing, China; 7 State Key Laboratory for Infectious Disease Prevention and Control and National Center for AIDS/STD Control and Prevention (NCAIDS), Chinese Center for Disease Control and Prevention, Beijing, China; Rush University, United States of America

## Abstract

**Objective:**

Three models for promoting male circumcision (MC) as a preventative intervention against HIV infection were compared among migrant worker populations in western China.

**Methods:**

A cohort study was performed after an initial cross-sectional survey among migrant workers in three provincial level districts with high HIV prevalence in western China. A total of 1,670 HIV seronegative male migrants were cluster-randomized into three intervention models, in which the dissemination of promotional materials and expert- and volunteer-led discussions are conducted in one, two, and three stage interventions. Changes in knowledge of MC, acceptability of MC, MC surgery uptake, and the costs of implementation were analyzed at 6-month and 9-month follow-up visits.

**Results:**

All three models significantly increased the participants’ knowledge about MC. The three-stage model significantly increased the acceptability of MC among participants and led to greatest increase in MC uptake. At the end of follow-up, 9.2% (153/1,670) of participants underwent MC surgery; uptake among the one-, two-, and three-stage models were 4.9%, 9.3%, and 14.6%, respectively. Multivariable Cox regression analysis showed that three-stage model was the most effective method to scale up MC, with RR = 2.0 (95% CI, 1.3-3.1, P=0.002) compared to the on-site session model. The two-stage intervention model showed no significant difference with either the on-site session model (RR=1.5, 95% CI, 0.92-2.4, P=0.12) or three-stage model (P=0.10).

**Conclusions:**

A three-stage intervention with gradual introduction of knowledge led to the significantly increase in MC uptake among migrant workers in western China, and was also the most cost-effective method among the three models.

## Introduction

Epidemiological evidence indicates that the epidemic of HIV/AIDS in China has seen a distinct increase in heterosexual transmission in recent years [1–3]. An estimated 740,000 people are living with HIV/AIDS in China, 27% of which are found in three western provinces and municipalities Xinjiang, Guangxi and Chongqing, which only contains 7.5% of China’s total population [4]. As well, in Chongqing, Guangxi and Yunnan province, the proportion of men infected with HIV via sexual contact increased from 7.3% in 2000 to 45% in 2009 [5]. Meanwhile, China has experienced an upsurge of labor force migration since the 1990s. There were an estimated 253 million domestic migrants in 2011, an increase by 4.4% from the 242 million migrants in 2010 [6]. Blue-collar workers comprised roughly 80% of this total, more than half of whom were men [6]. The migrants are recognized to be a bridge population for transmitting HIV and other sexually transmitted infections (STIs) to their spouses, since long separation from their spouses may lead them to purchase, and in some cases sell, sex while being away from home [7,8]. According to surveys in Guangxi and Sichuan in 2007, 16% of migrants had casual or commercial sex in the past year, only 35% of which were safe sex, and the rate of protected sex at last sex with their spouses was only 22% [5]. High-risk behaviors of migrants put both themselves and their spouses in rural homes at a high risk of HIV infection [8–10]. Thus, one goal outlined in the 2006–2010 National Five-Year Action Plan was that 70% of China’s migrant population should be reached by educational interventions on HIV/AIDS and should understand HIV transmission and prevention [11].

A systematic review and meta-analysis of randomized controlled trials in southern Africa has shown that MC reduced the risk of HIV infection by 38-66% over 24 months in heterosexual men [12–15]. As well, uncircumcised men have higher risk of acquiring genital ulcer disease than circumcised men [16]. MC may be more effective in preventing or controlling HIV transmission in some countries where HIV prevalence is high but MC rate is low, and where the route of transmission is predominantly heterosexual intercourse [17,18]. It was estimated that increased MC coverage could reduce HIV prevalence by up to 67% in a predominantly heterosexual population [19]. However, MC rates vary between countries and ethnicities [17]. Acceptance rates for MC are higher in the United States, Canada, the Middle East, Asian Muslim countries and some African countries, ranging between 20-80% to even almost universal [17]. In China, the estimated rate of MC practice is 2.7%, except for the Muslim minorities Hui and Uighur, which have higher rates due to religious traditions [6].

Our preliminary survey results showed that the rate of willingness to accept MC among male migrants in Guangxi, Xinjiang and Chongqing averaged 38.1% (manuscript in submission). Education about the benefits of MC for this population is therefore needed in western China. However, specific guidelines on conducting interventions among migrant workers have not yet been developed. Many routine HIV prevention programs in China currently include information on MC in one-time on-site education sessions during the annual Chinese New Year holidays, when many migrants return to their hometowns and are exposed to residency-based public health campaigns, but their effectiveness has not been systematically evaluated [11]. To explore better intervention models, further research comparing traditional methodology and new intervention strategies for scaling up MC is necessary. This study examined three intervention models at migrant work sites with increasing numbers of intervention phases, comparing their effectiveness and cost-effectiveness for promoting MC among migrant workers in high HIV prevalence regions in western China.

## Methods

### Study sites and participants

The study was part of a larger study which investigated the factors influencing Chinese males’ willingness to undergo circumcision [20]. This study was carried out from September 2009 to December 2010 in three high HIV prevalence provinces and municipalities in western China, Guangxi, Xinjiang and Chongqing, each of which are home to over 30,000 people living with HIV/AIDS (over 60,000 each for Guangxi and Xinjiang) [4]. Construction sites or mining sites that employed at least 200 workers were contacted, and three sites were selected for each district. All workers on site were evaluated for study eligibility, which includes male migrant workers aged 18-55 years, who lived away from the townships of their household registration, and planned to live in their workplaces for more than one year, regardless of their marital status. As part of the larger study, participants had completed a baseline cross-sectional survey on general knowledge of HIV/AIDS and willingness to accept MC as prevention for HIV transmission.

To be eligible for our intervention study, blood samples of all participants were screened by ELISA (enzyme linked immunosorbent assay) and RPR (Rapid Plasma Reagent) to confirm sero-negative status for HIV and syphilis. Urethral or penile swabs were taken if urethral discharge or genital ulcers were available. We excluded previously circumcised men and those who could not answer the questionnaires independently.

**Figure 1:**
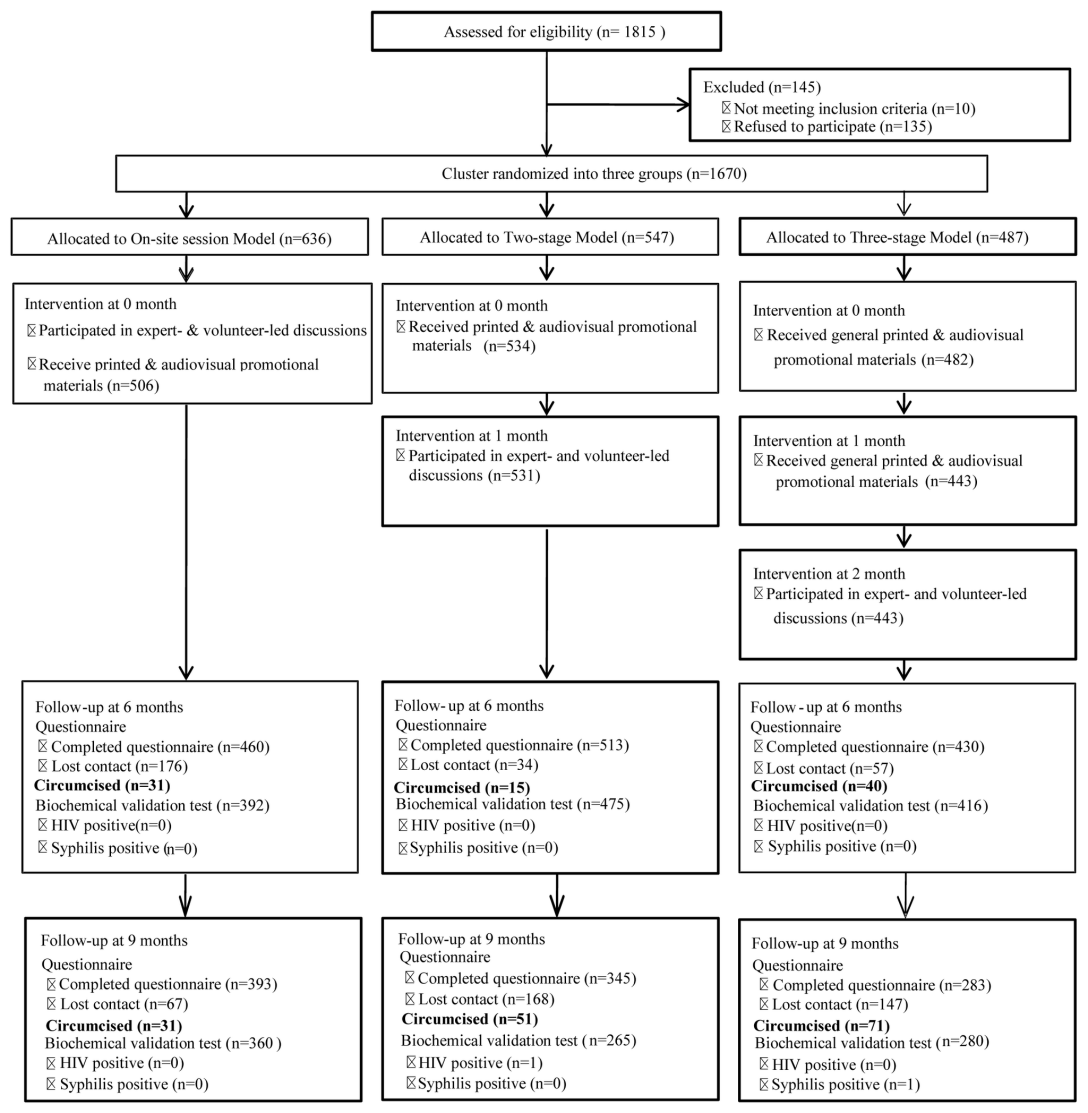
The flow chart of three intervention models. A total of 1,815 subjects participated in the preliminary survey that is part of a larger study on MC in China. Of these, 1,670 agreed to participate in this intervention study and were cluster-randomized by their work site to one of three intervention models. The standard model consisted a one-time on-site session, in which participants received special printed and audio-visual materials and participated in expert- and volunteer-led discussions. The two-stage model delivered special printed and audio-visual materials at 0 month, followed by expert- and volunteer-led discussions after at ~1 month interval. The three-stage model delivered general printed and audio-visual materials at 0 month, special printed and audio-visual materials at ~1 month, and expert- and volunteer-led discussions at ~2 months. Participants’ knowledge of MC, willingness to accept MC, number of MC surgeries undertaken, cost per uptake, and HIV/STIs infections were surveyed at 6-month and 9-month follow-up visits. Within each model, participants who did not receive a particular stage of interventions were considered lost to follow up and were discontinued from all next intervention stages. Only data with complete intervention and questionnaire records were eligible for analysis.

In total, 1,815 male migrant workers were invited to participate. Of these, 145 people were excluded, consisting of 135 who refused to be followed up, 5 who were HIV sero-positive, and 5 who were already circumcised. The resulting 1,670 male migrants enrolled were allocated to three different MC-related education intervention groups, through cluster randomization by work site. All subjects at the same work site received the same intervention. MC surgery was available free of charge at the local hospitals (nationally accredited at level 3A), where surgeons received MC-specific training and qualification assessments from the study researchers, and provided free postoperative treatment and consultation (Figure 1).

### Ethics Statement

We obtained written informed consent from each participant prior to their enrollment. Approval for this study, including study protocol and the circumcision program, was obtained from the Ethics and Human Subjects Committee of Guangxi Medical University.

### Intervention Models

The content of the intervention models included a series of general and special promotional media materials. The general printed texts are brochures and orientation panels named the “Handbook of MC as Protection Against AIDS Transmission”, which includes the scientific context of MC, MC and AIDS prevention, benefits of MC in reproductive and sexual health, do’s and don’ts during the perioperative period, and so on. The general audio-visual productions consist of flash animation and scenario educational films about how MC protects people from HIV infection. The special printed texts also consist of both brochures and orientation panels but are different in content, which presented knowledge on MC through experiences of medical university students who underwent MC, especially the psychological process of learning about and accepting MC. The special audio-visual productions had the same content as the special printed texts. In addition, the special promotional program included on-site sessions and discussions led by invited experts and university student volunteers.

Three intervention models were compared (Figure 1). The standard model used all intervention materials and held a one-time on-site session that included dissemination of materials and expert- and volunteer-led discussions. The two-stage model separated the dissemination of all materials from the on-site discussion sessions, with a 2-4 week interval between the stages. The three-stage model separated the distribution of general materials, the distribution of special materials, and the discussions into three stages, with a 2-4 week interval between every stage (Figure 1).

### Statistical analysis

All data were double entered into EpiData software (EpiData 3.1 for Windows; The EpiData Association, Odense, Denmark) and analyzed using SPSS for windows Version 19.0 (SPSS, Chicago IL). Descriptive statistics was generated for each variable corresponding to specific questions in the survey, including general characteristics and reasons for willingness or unwillingness to accept MC. Cost effectiveness analysis was performed on the total cost of each intervention model averaged over the number of MC surgeries undertaken by participants within the model. Cox-regression analysis was conducted to compare different types of interventions, with adjusting for demographic and migration characteristics and estimated relative risk (RR) with 95% confidence intervals. The baseline time was defined as the start point. The end point was either MC surgery uptake or the end of the second follow-up session, whichever came first. The incidences of HIV and syphilis infection in circumcised and non-circumcised subjects were compared using chi-squared test. All statistical tests were two-sided with significance level of *P* < 0.05. In addition, cost-effectiveness analysis was conducted for each model by calculating the cost of implementation (including promotional material cost, service charge, transportation expenses, and on-site investigation and intervention costs) for every subject who underwent MC surgery.

## Results

### Study population

A total of 1,670 male migrants were cluster-randomized by work site into three intervention groups: on-site session model (n = 487), two-stage intervention model (n = 547), and three-stage model (n = 636). At the sixth month after intervention, 460 (72.3%), 513 (93.8%), and 430 (88.3%) participants were followed up for each group respectively. At the ninth month of follow-up, 1,021 provided valid responses to the final questionnaire. The three models had no significant differences in self-reported religious affiliations, drug abuse history, and average scores of general knowledge about AIDS. There were statistically significant differences between the three models in terms of age, ethnicity, marital status, education level, place of origin, sexual history, and select STIs statuses, although their distribution showed generally consistent trends across the three models (Table 1).

**Table 1:** Baseline of characteristics of three models for scaling up MC among migrant workers in western China.

	On-site Session Model (n=636) (%)	Two-stage Model(n=547) (%)	Three-stage Model (n=487) (%)	χ^2^ (F)	*P*
Age (Mean, SD,years)	30.8±8.5	33.8±8.7	30.0±8.9	29.063	0.000
<25	184 (28.9)	96 (17.6)	179 (36.8)		
25^~^35	247 (38.8)	206 (37.7)	162 (33.3)		
>35	205 (32.2)	245 (44.8)	146 (30.0)	57.105	0.000
Ethnicity				10.770	0.029
Han	423 (66.5)	387 (70.7)	336 (69.0)		
Zhuang (Chuang)	205 (32.2)	145 (26.5)	134 (27.5)		
Others	8 (1.3)	15 (2.8)	17 (3.5)		
Marital status				24.658	0.000
Married/Cohabitating	418 (65.7)	413 (75.5)	300 (61.6)		
Not married	218 (34.3)	134 (24.5)	187 (38.4)		
Education				20.589	0.000
Primary school/Illiteracy	35 (5.5)	43 (7.9)	59 (12.1)		
Middle school	354 (55.7)	306 (55.9)	231 (47.4)		
High school above	247 (38.8)	198 (36.2)	197 (40.5)		
Religion				14.667	0.066
None	534 (84.0)	438 (80.1)	404 (83.0)		
Islam	5 (0.8)	4 (0.7)	1 (0.2)		
Taoism	15 (2.4)	26 (4.8)	12 (2.5)		
Buddhism	67 (10.5)	74 (13.5)	60 (12.3)		
Others	15 (2.4)	5 (0.9)	10 (2.1)		
Original residence				11.597	0.021
Guangxi	237 (37.3)	174 (31.8)	160 (32.9)		
Chongqing	180 (28.3)	190 (34.7)	180 (37.0)		
Xinjiang	219 (34.4)	183 (33.5)	147 (30.1)		
Average scores on general AIDS knowledge				4.690	0.096
≦Mean	295 (46.4)	283 (51.7)	253 (52.0)		
﹥Mean	341 (53.6)	264 (48.3)	234 (48.0)		
Drug abuse				2.513	0.285
Yes	9 (1.4)	3 (0.5)	7 (1.4)		
No	627 (98.6)	544 (99.5)	480 (98.6)		
Sexual histories				6.964	0.031
Yes	491 (77.2)	452 (82.6)	374 (76.8)		
No	145 (22.8)	95 (17.4)	113 (23.2)		
History of Related STIs					
Concealed penis	3 (0.5)	8 (1.5)	4 (0.8)	3.289	0.193
Phimosis inflammation	20 (3.1)	11 (2.0)	22 (4.5)	5.270	0.072
Genital ulcer	2 (0.3)	5 (0.9)	10 (2.1)	8.364	0.015
Dysuria	11 (1.7)	22 (4.0)	22 (4.5)	8.086	0.018
Pyuria	4 (0.6)	11 (2.0)	10 (2.1)	5.252	0.072

### Change in knowledge on MC at six-month and nine-month follow ups

The models were compared for their effectiveness in changing participants’ knowledge, attitudes and practices (KAP) regarding MC in follow-up sessions at the sixth month and ninth month after the interventions’ completion. Results showed that all three models led to statistically significant increases in KAP scores at both follow-up times compared to baseline, for almost every category and item of the assessment (Table 2). The two-stage intervention model was the most effective in changing the participants’ knowledge on MC, with the greatest increases in general knowledge on MC, potential surgical complications, as well as the effect of MC on sexual functions (Table 2).

**Table 2:** Changes in knowledge on MC at 6- and 9-month follow up sessions among migrant workers in western China.

	On-site Session Model			Two-stage Model			Three-stage Model		
	Baseline (%) (n=636)	6 months (n=460)	9 months (%) (n=393)	Baseline (%) (n=547)	6 months (n=513)	9 months (%) (n=345)	Baseline (%) (n=487)	6 months (%) (n=430)	9 months (%) (n=283)
Knowledge of MC									
Phimosis/prepuceredundant	311 (48.9)	236 (51.3)	273 (69.5)**	241 (44.1)	378 (73.7)**	183 (53.0)**	221 (45.4)	224 (52.1)*	155 (54.8)*
Prevention of genital inflammation & tumors	166 (26.1)	227 (49.3)**	239 (60.8)**	112 (20.5)	356 (69.4)**	169 (49.0)**	120 (24.6)	236 (54.9)**	142 (50.2)**
Prevention of AIDS & STIs	131 (20.6)	337 (73.3)**	273 (69.5)**	104 (19.0)	385 (75.0)**	188 (54.5)**	119 (24.4)	301 (70.0)**	160 (56.5)**
Protect sexual partner	235 (36.9)	282 (61.3)**	265 (67.4)**	173 (31.6)	321 (62.6)**	185 (53.6)**	158 (32.4)	262 (60.9)**	165 (58.3)**
Increase sexual satisfaction	150 (23.6)	165 (35.9)**	194 (49.4)**	134 (24.5)	223 (45.4)**	127 (36.8)**	119 (24.4)	174 (40.5)**	114 (40.3)**
Improve physical appearance	67 (10.5)	66 (14.3)	77 (19.6)**	47 (8.6)	69 (13.5)*	53 (15.4)**	46 (9.4)	68 (15.8)**	42 (14.8)*
No knowledge	248 (39.0)	4 (0.9)**	17 (4.3)**	248 (45.3)	4 (0.8)**	32 (9.3)**	201 (41.3)	7 (1.6)**	24 (8.5)**
Knowledge of MC complication									
Pain	176 (27.7)	245 (53.3)**	241 (61.3)**	108 (19.7)	343 (66.9)**	175 (50.7)**	129 (26.5)	201 (46.7)**	162 (57.2)**
Bleeding	117 (18.4)	199 (43.3)**	216 (55.0)**	86 (15.7)	271 (52.8)**	123 (35.7)**	87 (17.9)	179 (41.6)**	119 (42.0)**
Wound infections	214 (33.6)	236 (51.3)**	212 (53.9)**	165 (30.2)	324 (63.2)**	169 (49.0)**	138 (28.3)	223 (51.9)**	131 (46.3)**
No knowledge	346 (54.4)	103 (22.4)**	6 (1.5)**	321 (58.7)	93 (18.1)**	7 (2.0)**	295 (60.6)	120 (27.9)**	7 (2.5)**
Effect on sexual functions									
Improved	149 (23.4)	139 (30.2)*	182 (46.3)**	129 (23.6)	236 (46.0)**	114 (33.0)**	104 (21.4)	175 (40.7)**	127 (44.9)**
Declined	52 (8.2)	47 (10.2)	19 (4.8)*	30 (5.5)	21 (4.1)	40 (11.6)**	31 (6.4)	37 (8.6)	23 (8.1)
No effect	102 (16.0)	148 (32.2)**	105 (26.7)**	65 (11.9)	121 (23.6)**	92 (26.7)**	58 (11.9)	100 (23.3)**	57 (20.1)**
No knowledge	329 (51.7)	126 (27.4)**	87 (22.1)**	323 (59.0)	135 (26.3)**	99 (28.7)**	294 (60.4)	118 (27.4)**	76 (26.9)**

* P<0.5; ** P<0.01. As compared to the baseline value within the intervention model.

### Acceptability of MC and uptake of MC among three intervention models

We assessed the participants’ reported willingness to accept MC as well as actual MC uptake following the interventions. Uptake of MC surgery was statistically different among the three intervention models, with the three-stage model being the most effective (Table 3). A total of 153 cases of circumcision surgery were performed at the end of follow-up, 71 (46%) of which resulted from the three-stage model; the two-stage model resulted in 51 cases (33%) and the on-site session model resulted in 31 cases (20%). Intervention resulted in an overall MC prevalence of 9.2% (95% CI, 7.8-10.7%); prevalence was 14.6% (95% CI, 11.4-18.4%) in the three-stage model, 9.3% (95% CI, 6.9-12.3%) in the two-stage intervention model, and 4.9% (95% CI, 3.3-6.9%) in the on-site session model. Although the interventions led to changes in surgery uptake, they did not significantly alter the participants’ self-reported willingness to accept MC, and the effects of different models were also not significantly different (P=0.065). The on-site session model led to a 2.4% increase in MC acceptability; the two-stage model, 5.6%; and the three-stage model, 3.8%.

**Table 3:** Changes in willingness to accept MC and in uptake of MC surgery at 9-month follow up after three intervention models in migrant workers in western China.

	Baseline willingness to accept MC % (n_0_/N)	Willingness to accept MC at 9 months ^a^ % (n_0_/n)	Uptake of MC surgery at 9 months^b^ % (n_0_/N)
On-site session Model	35.5 (226/636)	37.9 (149/393)	4.9 (31/636)
Two-stage Model	40.8 (223/547)	46.4 (160/345)	9.3 (51/547)
Three-stage Model	39.0 (190/487)	42.8 (121/283)	14.6 (71/487)
χ^2^	3.573	5.464	31.239
P	0.168	0.065	0.000

a. Data for willingness to accept MC is partially missing due to non-response at follow-up.

b. Uptake of MC use the ITT (intention to treat) analysis. n_0_ is defined as the new event in the follow up; N is the total number of participants baseline; n is the total number at the end of follow up.

Cox proportional relative risk (RR) regression model was performed to compare the effects of different intervention models on the uptake of MC in migrant workers (Table 4). Results showed that the three-stage model was most effective at promoting MC uptake, being approximately twice as effective as the on-site session model (*RR*= 2.0, 95% CI, 1.3-3.1, *P*=0.01). The two-stage intervention model showed no significant effect when compared with either the on-site session model (RR=1.5, 95% CI 0.92-2.4, P=0.12) or the three-stage model (P=0.10).

**Table 4:** Cox regression model to compare the effect of three models among migrant workers.

	RR*	95% CI	*P*
On-site session Model^#^	1	—	—
Two-stage Model^#^	1.486	0.924-2.388	0.102
Three-stage Model^#^	2.021	1.303-3.134	0.002

Note: * Cox-regression analyses were conducted to compare different kinds of interventions, and adjusting for demographic (age, ethnicity, marital status and education) and migration characteristics (original residence), estimated RR (relative risk) with 95% CI (confidence interval). ^#^ The baseline time was defined as the start point. The end point was either MC surgery uptake or the end of the second follow-up session, whichever came first.

### Cost and Cost-effectiveness analysis

The costs and cost-effectiveness of the intervention models were compared. The input cost was defined as the total cost of the model divided by the number of recipients who received the model, while the cost effectiveness was assessed by dividing the total cost with the number of participants who uptook MC surgery due to the model’s interventions. The total costs included all expenses for developing the promotional materials, performing services, training, travel, and on site investigation and intervention. The input cost for the on-site session model was 45.3 RMB ($7.11 USD); for the two-stage model, 74.4 RMB ($11.68 USD); and for the three-stage model, 109.8 RMB ($17.24 USD). On the other hand, the cost per successful MC uptake for the on-site session model was highest at 929 RMB ($145.76) per case; the two-stage intervention model cost 798 RMB ($125.20) per case; and the three-stage model, 753 RMB ($118.14) per case. The cost per successful uptake showed that the three-stage model was significantly more cost effective than either the two-stage or the on-site session model. One-way sensitivity analysis for the cost per MC uptake showed that varying the most significant cost contributor by up to 10% did not change the nature of the cost effective comparisons: the three-stage model remained significantly more cost effective than either the two-stage or the on-site session model.

### HIV/STD infection rate

The rates of HIV and syphilis infections acquired during the follow-up period were also compared between subjects who underwent MC and those who did not. HIV and syphilis incidences were zero among those who had undergone MC, while those who did not undergo MC had one case (incidence: 0.21 per 100 person-years) for both diseases.

## Discussion

Circumcision has been shown in RCTs to be one of the most effective ways to reduce men’s risk of HIV infection through heterosexual contact [13–15]. Compared to other prevention measures such as condom use, MC is a one-time intervention with lifelong benefits. Promoting MC among China’s male migrants may therefore have important implications for HIV/AIDS prevention, particularly in the developing Western regions where safe sex practices are not the norm and access to condoms may be unreliable [8,21,22]. In this study, we found that work-site based interventions improved knowledge about MC among Western Chinese migrant workers regardless of the intervention model employed, while the three-stage model was the most effective in promoting MC surgery uptake as well as the most cost-effective.

Our study resulted in an overall MC prevalence of 9.2% among participants, equivalent to 16 MC operations per month, and a prevalence of 14.6% among those who received the third intervention model. These are low compared with sub-Saharan Africa, where the median proportion of uncircumcised men willing to become circumcised was 65% (range 29–87%) across nine countries and thirteen studies [18]. However, unlike these regions where male circumcision is frequently conducted as a traditional rite of passage, MC is not a traditional practice in China [23-25]. The estimated prevalence of MC practice in China is 2.7% [6]. Our results therefore indicated a significant increase in MC uptake. Nonetheless, the effectiveness of MC as a preventative tool against HIV transmission also depends on population HIV prevalence. HIV prevalence was low among both our study subjects (0.098% after nine months of follow up, 95% CI 0.0025-0.55) and in China overall (0.057% in 2009, 95% CI, 0.042-0.071%) [4], which suggests that preventative effects may be limited. But attempted to make the case that China’s 253 million (and growing) migrant workers are a huge potential risk pool for HIV infections [6]. Migrant workers exhibit demographic characteristics that would put them at high risk for heterosexual HIV transmission, such as unstable family and work situations, ease of access to high-risk (including commercial) sex, and lack of access to China’s permanent residency-based HIV treatment and prevention programs [9]. Further evaluations are needed to determine the effectiveness and feasibility of promoting MC as a means to reduce HIV transmission among migrant workers and other at-risk populations.

Our results indicated that a multi-staged intervention that gradually reinforces knowledge from promotional materials to peer and educator led discussions is the most effective model to promote MC in China. The involvement of peers, partners, and medical college students likely played a key role in encouraging participants to retain and internalize knowledge on MC and to take action on seeking MC surgery. As well, while the three intervention models did not differ in their ability in changing the participants’ willingness towards MC, the three-stage model was significantly more effective in promoting MC surgery uptake. This suggests that self-reported attitude towards MC is not strongly correlated with actual behavior in this population.

Our study is subject to some limitations. We conducted two follow-ups after baseline, at six months (1,412 samples, response rate 87%, with 1,403 completed questionnaires) and at nine months (1,021 samples, response rate 61%). The second time point saw a high loss to follow up in Xinjiang, where severe winter weather conditions and social unrest led to low response rates, while Guangxi and Chongqing had 79% follow up. As well, while the prevalence of HIV and syphilis among uncircumcised men was higher than in circumcised men, this was not statistically significant. This may be due to a small sample size or an insufficiently long observation time. Nonetheless, our results demonstrate that a promotion model for MC consisting of three stages and progressively interactive interventions can increase circumcision among immigrant workers in western China. To explore the effectiveness of MC on reducing sexual transmission of HIV among migrant workers, future studies having an expanded sample size, longer postoperative observation time, and improved quality of pre- and post-operative counseling are necessary.
